# Change in upper limb function in people with multiple sclerosis treated with nabiximols: a quantitative kinematic pilot study

**DOI:** 10.1007/s10072-022-06456-3

**Published:** 2022-10-19

**Authors:** Massimiliano Pau, Micaela Porta, Gabriella Spinicci, Jessica Frau, Lorena Lorefice, Giancarlo Coghe, Eleonora Cocco

**Affiliations:** 1 Department of Mechanical, Chemical and Materials Engineering, University of Cagliari, Piazza d’Armi, 09123 Cagliari, Italy; 2 Multiple Sclerosis Centre, Binaghi Hospital, ATS Sardegna, Department of Medical Sciences and Public Health, University of Cagliari, Cagliari, Italy

**Keywords:** Multiple sclerosis (MS), Upper limb, Spasticity, Nabiximols, Dexterity, Kinematics, Hand to mouth

## Abstract

**Objectives:**

Nabiximols represents an increasingly employed add-on treatment option for spasticity in people with multiple sclerosis (PwMS) who either were unresponsive or reported excessive adverse reactions to other therapies. While several studies performed in the last decade demonstrated its effectiveness, safety, and tolerability, few quantitative data are available on the impact on motor dysfunctions. In this open-label, not concurrently controlled study, we aimed to assess the impact of a 4-week treatment with nabiximols on upper limb functionality.

**Methods:**

Thirteen PwMS (9 female, 4 male) with moderate-severe spasticity underwent a combination of clinical tests (i.e., Box and Block, BBT and Nine-Hole Peg test, 9HPT) and instrumental kinematic analysis of the “hand to mouth” (HTM) movement by means of optical motion capture system.

**Results:**

After the treatment, improvements in gross and fine dexterity were found (BBT + 3 blocks/min, 9HPT − 2.9 s, *p* < 0.05 for both cases). The kinematic analysis indicated that HTM movement was faster (1.69 vs. 1.83 s, *p* = 0.05), smoother, and more stable. A significant reduction of the severity of spasticity, as indicated by the 0–10 numerical rating scale (4.2 vs. 6.3, *p* < 0.001), was also observed.

**Conclusion:**

The findings from the present pilot study suggest that a 4-week treatment with nabiximols ameliorates the spasticity symptoms and the overall motor function of upper limb in PwMS with moderate-severe spasticity. The use of quantitative techniques for human movement analysis may provide valuable information about changes originated by the treatment in realistic upper limb motor tasks involved in activities of daily living.

## Introduction

One of the most distinctive features of multiple sclerosis (MS), a chronic inflammatory disease of the central nervous system (CNS) which affects 2.8 million individuals worldwide [1], is the accumulation of deficits and progressive disability, which are consequent to demyelination and axonal loss within the central nervous system (CNS) and express as visual impairment, cognitive dysfunction, spasticity, motor weakness, incoordination, fatigue, bowel and bladder dysfunction, and impaired mobility [2]. In particular, the progressive deterioration across the clinical course of the disease of basic motor functions, including postural control and ambulation [3, 4], often compromises the ability to optimally perform many activities of daily living (ADL) thus severely limiting independence and quality of life [5].

While lower limb motor impairments are pivotal in determining the degree of disability of people with MS (PwMS) (as indicated by their central role in the Expanded Disability Status Scale, EDSS) and great attention is devoted to their management, it is noteworthy that a relevant percentage of PwMS (estimated between 50 and 80% [6, 7]) also complain of upper limb dysfunctions, which are multifactorial in nature and include weakness, spasticity, ataxia, tremor, sensory loss, and pain. These usually manifest under the form of reduced manual dexterity (both gross and fine), slowness of movements, dysmetria, and clumsiness [8, 9] and may negatively affect a wide range of ADL including manipulation of objects, writing, self-care skills (i.e., dressing, grooming, etc.) as well as occupational/school performance [10].

Among the factors previously listed, it is noticeable that the management of upper extremity spasticity, despite the non-negligible number of PwMS who suffer from it [11], appears challenging and mostly unexplored. Indeed, scarce data are available not only on the real extent of the phenomenon but also as regards the adopted therapeutic approaches. In particular, specific evidence on the effectiveness of treatments based on the recommended anti-spasticity oral therapies (i.e., baclofen, tizanidine, gabapentin, etc., primarily employed in PwMS to restore gait functionality) on upper limb function is limited and only rarely supported by quantitative biomechanical assessments [12].

Since 2010, nabiximols (Sativex®), an oromucosal spray whose active principle is a combination of 9-delta tetrahydrocannabinol (THC) and cannabidiol (CBD), was approved in Europe as an add-on treatment option for spasticity in PwMS who were unresponsive to other treatments. Its specific formulation, particularly as regards to the fraction of CBD of the total active moiety, was designed to avoid the typical side effects associated with THC that characterize other cannabis-derived drugs [13]. The results of more than a decade of clinical evaluation and post-approval daily clinical practice studies about effectiveness and safety in the management of MS-related spasticity show that nabiximols represents a suitable therapeutic option especially in terms of PwMS’ and caregivers’ perception and of patient-reported outcome measures like the 0–10 numerical rating scale (NRS) and visual analog scale (VAS) [14–16]. On the other hand, it should be remarked that available data about its actual effects on motor function, especially objectively and instrumentally assessed, are limited. In particular, few studies have been carried out using state-of-the-art quantitative techniques for human movement analysis, which have been recognized useful to characterize altered movement in MS [17] and, especially in combination with surface electromyography, suitable to perform objective and accurate assessment of the effects of treatments to reduce muscle overactivity on the movement to be improved [18]. It should also be noted that such studies were targeted on gait [19, 20] while, to our knowledge, possible positive effects of nabiximols on upper extremities movements have never been investigated.

On the basis of the aforementioned considerations, here we pursued a pilot study aimed to quantitatively and objectively assess the effects of nabiximols on upper limb functioning of PwMS by analyzing several kinematic features of the “hand-to-mouth” (HTM) functional task. Such movement is considered well representative of important ADLs like eating and drinking [21] and was selected in previous studies which investigated upper limb functions in individuals affected by neurological diseases [22–25] including MS [26]. The results might have a relevant impact on the overall assessment of the effectiveness of the treatment, firstly because the instrumental kinematic analysis may reveal changes in UL function not easily detectable with other clinical tests, but also because the HTM task, being commonly performed on a daily basis, should better reflect the actual potential of the treatment in terms of increased ability to perform activities essential for the independence of PwMS.

## Materials and methods

### Participants

The study was carried out at the Regional MS Center of Cagliari (Sardinia, Italy) and at the Laboratory of Biomechanics and Industrial Ergonomics of the University of Cagliari (Cagliari, Italy). The participants were selected among PwMS considered eligible for using nabiximols based on their clinical history. Inclusion criteria were as follows: a diagnosis of MS according to the 2017 McDonald criteria [27]; absence of clinical or neuroradiological relapses from at least 6 months before study entry; presence of moderate to severe spasticity (assessed through a 0–10 numeric rating scale, NRS [28] ⩾ 4); lack of response to common and ongoing spasticity treatments; absence of concomitant severe cardiovascular diseases; no prior or current psychiatric diseases; no current use of cannabis and/or other psychoactive drugs; and the ability to take nabiximols according to medical judgment and the Italian Drugs Agency (Agenzia Italiana del Farmaco, AIFA) approved label-related criteria.

Immediately after the nabiximols prescription, PwMS were offered to enter the study. The baseline assessment (*T*_0_), which was performed prior to the assumption of the first dose of nabiximols, included a neurological evaluation and an EDSS assessment performed by a Neurostatus [29] certified neurologist, the measurement of spasticity severity from PwMS perspective through a 0–10 NRS, and two clinical tests widely employed to assess gross and fine manual dexterity, namely, the Box and Block test (BBT) and the Nine-Hole Peg test (9HPT). Moreover, participants underwent the kinematic evaluation of the upper limb function during the execution of the HTM task, as described in detail below. The treatment was started with gradual titration until the optimal dose was reached. PwMS were required to track their dosing patterns through a diary to ensure a satisfactory compliance with the treatment. After 4 weeks of stable treatment, they were called to undergo a second visit (*T*_1_) during which EDSS, 0–10 NRS, and upper limb function were retested.

The study, which was conducted according to the World Medical Association Declaration of Helsinki principles, received formal approval by the ethics committee of ATS Sardegna (protocol number 198/2019/CE). All participants signed an informed consent agreeing to participate.

### The “hand to mouth” task

To perform the “hand to mouth” (HTM) task, PwMS were required to comfortably sit on a chair positioned in front of a table over which they placed their hands palms down. The table height was adjusted in such a way to ensure that both shoulders and wrists assumed a neutral position, with the elbows flexed at approximately 90° and the forearm prone [22–24, 26, 30]. Then, participants were instructed to complete the HTM task, at self-selected pace, according to the following sequence: from the starting position, following an acoustic signal, they moved their hand towards the face until the fingertips touched their mouth, then returned it to the starting position. After a brief familiarization phase, participants performed three repetitions of the movement with each limb. The six trials were acquired, and the average value of right and left limb separately considered for the subsequent processing.

### Kinematic data acquisition and processing

Data acquisition was carried out by means of an optical motion capture system equipped with 8 infrared cameras set at a sampling rate of 120 Hz (SMART-D, BTS Bioengineering, Milan, Italy). Retro-reflective markers (14 mm diameter) were positioned bilaterally on the acromion, lateral humeral epicondyle, ulnar and radial styloid processes, on second metacarpal head and on the fingertip to identify the position and orientation of, respectively, the upper arm, forearm, and hand. The head and trunk positions were estimated through markers positioned respectively on the zygomatic, nasion processes and mouth under the inferior lip (head), right and left acromion, clavicular notch and spinous processes of C7 and T8 vertebrae (trunk) according to standardized protocols previously employed for similar purposes [31, 32]. The three-dimensional trajectories of the markers, acquired during the HTM task execution, were processed using a dedicated custom routine developed under the Smart Analyzer environment (BTS Bioengineering, Milan, Italy). In particular, according to previous similar studies [21, 24, 26, 33] the whole HTM movement was segmented into three phases as follows:

- Going phase, which identifies the hand movement from the table to the mouth
- Adjusting phase, dedicated to precisely locating the mouth
- Returning phase, during which the hand is moved back to the initial position

In particular, the beginning of the going phase was identified as the point in time at which the linear velocity of the fingertip marker exceeded 20% of the peak velocity [34] and, similarly, its end corresponded as the time in which the velocity dropped below 20% of the peak velocity. The same threshold was adopted to identify the returning phase and thus the adjusting phase was identified as the time period in which the fingertip marker velocity was always below the 20% of the peak velocity value.

Each participant’s performance was further quantitatively characterized by calculating:

- Total movement duration (s).
- Time needed to complete going, adjusting, and return phases (s).
- Index of curvature: which is a dimensionless parameter obtained by dividing the length of the 3D trajectory of the marker placed on the fingertip and the linear distance between its initial position and the target (i.e., mouth [24, 30]). This parameter is representative of movement smoothness during the going phase and its interpretation is quite straightforward (i.e., the lower, the smoother the movement).
- Number of movement units: that is, the number of velocity peaks that exceeded the 10% of the maximum velocity calculated across the entire task. Such value is representative of repeated accelerations and decelerations during movement performance and reflects efficiency and smoothness of movement [35]. An ideal perfectly smooth movement would have only one movement unit and reference values for healthy individuals approximately lie in the range 1.1–1.2 [36, 37].
- Frequency of changes in direction (Hz): this is another measure of smoothness which characterizes the finger displacements associated to the possible presence of tremor [30, 38]. To calculate this parameter, we filtered the fingertip marker trajectory using a band-pass filter (2–10 Hz) to separate voluntary movements (0–2 Hz) from tremor (2–12 Hz). Previous studies performed on PwMS indicated that, during the HTM task, they exhibit higher values of this parameter with respect to unaffected individuals [26].

While the listed parameters are the most commonly employed to characterize the HTM performance from a kinematic point of view, it is noteworthy that the employed marker set also allows to obtain data about the angular trends at shoulder, elbow, and wrist joints during the movement execution. However, for the sake of brevity, such data will not be presented here.

### Statistical analysis

A preliminary independent sample *t*-test was carried out to assess possible differences between left and right limb of each subject at baseline, and no significant differences were found for any of the variables of interest. Thus, the mean value calculated across the six trials performed by both limbs was considered representative of each participant.

Following a testing of the variables for normality (using the Shapiro–Wilk test) and homogeneity of variances (Levene’s test), if such assumptions were met the existence of possible differences associated with the treatment on performance of clinical tests, kinematic parameters of HTM and NRS score was assessed using one-way analysis of variance for repeated measures (RM-ANOVA) considering time (*T*_0_/*T*_1_) as independent variable and the six HTM parameters previously listed as dependent variables. Where necessary (i.e., variables not normally distributed), the Friedman test was employed. In all cases, the level of significance was set at *p* = 0.05. All analyses were performed using the IBM SPSS Statistics v. 20 software (IBM, Armonk, NY, USA).

## Results

Thirteen PwMS (4 male and 9 female) were enrolled between March 2021 and February 2022. Their main baseline anthropometric and clinical features are reported in Table 1.

**Table 1:** Anthropometric and clinical features of participants. Continuous variables are expressed as mean (SD). Categorical variables are expressed as counts

Participants (M, F)	13 (9F, 4M)
Age (years)	51.2 (11.8)
Body mass (kg)	61.8 (14.1)
Height (cm)	163.5 (7.9)
EDSS score	5.4 (1.6)
Type of MS	11 SP, 1 RR, 1 PP

*EDSS*, expanded disability status scale; *MS*, multiple sclerosis;* PP*, primary progressive; *RR*, relapsing–remitting; *SP*, secondary progressive

The mean (SD) NRS of spasticity was 6.3 (1.3) at baseline and was statistically significantly reduced to 4.1 (1.3) after 4 weeks of treatment (*p* < 0.001). All the participants were considered responders to nabiximols according to the criteria proposed by Novotna et al. (i.e., −  ≥ 20% reduction in the spasticity 0–10 NRS score [39]). The mean dose of nabiximols assumed by PwMS was 5.6 (1.8) sprays per day. No participant abandoned or required treatment withdrawal and no adverse events were referred by the patients.

The results of the clinical tests and the kinematic parameters of HTM calculated before and after the treatment are summarized in Table 2.

**Table 2:** Changes in clinical and kinematic parameters before and after the treatment observed in the 13 PwMS who participated in the study. Values are expressed as mean (SD)

	Variable	*T*_0_	*T*_1_	*p* value
Clinical parameters	0–10 NRS spasticity	6.3 (1.3)	4.2 (1.3)	< 0.001*
	Box and Block test (number of blocks)	49.0 (7.8)	52.0 (9.5)	0.058
	Nine-Hole Peg test (s)	30.4 (6.4)	27.5 (5.7)	0.010*
Kinematic parameters hand-to-mouth task	Total movement duration (s)	1.83 (0.43)	1.69 (0.32)	0.050*^†^
	Going phase (s)	0.76 (0.14)	0.71 (0.14)	0.036*
	Adjusting phase (s)	0.27 (0.33)	0.24 (0.20)	0.601
	Returning phase (s)	0.80 (0.10)	0.75 (0.10)	< 0.001*^†^
	Index of curvature	1.09 (0.04)	1.09 (0.05)	0.863
	Number of movement units	6.51 (4.0)	4.16 (2.6)	0.027*
	Frequency of changes in direction (Hz)	4.99 (1.26)	4.73 (1.66)	0.050*^†^

The symbol * denotes a statistically significant difference between *T*_0_ and *T*_1_; the symbol ^†^ indicates a non-parametric test

As regards the clinical tests for gross and fine manual dexterity, ANOVA revealed a significant difference between *T*_0_ and *T*_1_ on the time required to perform the 9HPT, which was reduced by approximately 10% [*F*(1,25) = 7.80, *p* = 0.010], while in case of BBT, although the number of transferred blocks was increased at *T*_1_ compared to *T*_0_, statistical significance was not attained [*F*(1,25) = 3.93, *p* = 0.058].

The results of the kinematic analysis of the HTM task show a trend of general improvement in terms of both speed and smoothness. After the treatment, participants required a reduced time needed to complete the task (1.69 vs. 1.83 s, χ^2^ = 3.85, *p* = 0.05) due to the temporal shortening of all sub-phases. In particular, for the going phase [*F*(1,23) = 4.91, *p* = 0.036] and for the return phase (χ^2^ = 12.46, *p* < 0.001). Moreover, they also exhibited increased smoothness of movement as indicated by the reduced number of movement units [*F*(1,25) = 5.50, *p* = 0.027] and frequency of change in direction (χ^2^ = 3.86, *p* = 0.05).

## Discussion

The present pilot study was aimed at verifying the possible effects originated by 4-week nabiximols treatment on upper limb functioning of PwMS with moderate-severe spasticity. In particular, such assessment was carried out both from a clinical point of view (by investigating gross and fine manual dexterity with tests widely employed in MS) and by analyzing the kinematic parameters associated with a functional task (i.e., HTM) obtained using quantitative techniques for movement analysis, following an approach previously employed in several studies aimed at exploring the effect of nabiximols on gait performance [19, 20]. Indeed, the choice of HTM to investigate potential changes in upper limb functioning was made, other for its excellent capability to reproduce important daily tasks (i.e., eating and drinking) to obtain more accurate information about the actual degree of transferability of the possible improvement consequent to the therapy on ADL. This can add valuable information on the impact of the treatment on every day’s life of PwMS, which is likely to effectively integrate those related to self-perception of the spasticity (i.e., 0–10 NRS), quality of life scales, Ashworth scale score, Barthel Index, etc.

The results of the kinematic analysis suggest that the 4-week treatment had a positive impact on the ability of the tested PwMS to perform the HTM task. In particular, the overall time required to complete the task, which at the baseline was 1.83 s (in line with what previously observed in similar studies on PwMS [26]), after the treatment was found reduced by approximately 10%. Such value summarizes a consistent shortening of all the three sub-phases, which was found statistically significant for going and return phase. The observed changes in the number of movement units and in the frequency of changes in direction, taken together, suggest that the movement is smoother, meaning that PwMS approaches the target performing less corrections and with increased stability and accuracy.

It was also interesting to observe that the improvements associated with the treatment are not restricted to the kinematic parameters of HTM but also involve the gross and fine manual dexterity performance, thus indicating that the observed effects are not task-specific but are likely to extend to the overall upper limb functioning. In particular, for the BBT the observed increase was, on average, of 3 blocks/min (+ 6%) while for the 9HPT the time necessary to complete the test reduced by 2.9 s (− 10%). However, it should be noted that both changes, although significant, remain below the threshold identified as representative of a clinically meaningful difference (which are 6 blocks/min for BBT and 15–20% for the 9HPT [40, 41]).

Summarizing, the integration of the objective instrumental data and the clinical assessment indicates a certain degree of improvement in the upper limb functioning induced by nabiximols. At the same time, the study confirmed the subjective perception of the reduction of the severity of the spasticity symptoms (together with other positive aspects associated with sleep quality and bladder function) that were, so far, the main outcomes considered in most previous studies of nabiximols in PwMS. Even though, unfortunately, there is scarcity of similar data in the literature, our findings appear somehow consistent with those reported in the review by Arroyo et al. [42], which highlighted that PwMS who received nabiximols reported improvements in daily activities performance, especially those like washing, dressing, and transferring which involve a significant use of upper limbs.

From a clinical point of view, the findings of the present study are of some relevance since they suggest that the objective kinematic analysis can effectively integrate the results of the clinical tests to provide a more realistic and comprehensive view of the possible positive effects associated with the nabiximols treatment. In fact, while conventional tests like 9HPT and BBT are still necessary to evaluate any pharmacologic treatment targeted to UL, considering the large amount of existing data on PwMS that can be used as reference, the data originated by the instrumental analysis of HTM is likely to reflect the actual impact of the treatment under more ecological conditions.

Some limitations of the study should be acknowledged: at first, given the nature of the pilot study, the sample here tested was small and thus generalization of the results is limited. Secondly, even though the kinematic analysis of HTM is objective and hardly influenced by voluntary behavior of the participants, the lack of a control group did not allow to assess the possible impact of placebo effects. Third, the follow-up time (1 month) might be too short to fully capture the improvement possibilities of the treatment, as the antispastic effect of nabiximols has been shown to increase up to the third month [39, 43, 44]. At last, even though the HTM task is well representative of important ADL, further studies should be carried out considering an extended set of gross and fine upper limb movements.

## Conclusion

The findings emerged from the present pilot study, carried out by employing an approach based on the combination of clinical measures of gross and fine dexterity and instrumental parameters associated with the HTM task, suggest that a 4-week treatment with nabiximols ameliorates both spasticity and the overall motor function of upper limb in PwMS with moderate to severe spasticity at the baseline. The use of state-of-the-art techniques for human movement analysis, which have already been successfully employed particularly to investigate gait, may provide valuable information about changes in realistic upper limb motor tasks involved in ADL, thus contributing to better understand the actual transferability of the possible improvements observed in other clinical measures to actual “real-life” situations.
